# Identification of key peptidoglycan hydrolases for morphogenesis, autolysis, and peptidoglycan composition of *Lactobacillus plantarum* WCFS1

**DOI:** 10.1186/1475-2859-11-137

**Published:** 2012-10-15

**Authors:** Thomas Rolain, Elvis Bernard, Pascal Courtin, Peter A Bron, Michiel Kleerebezem, Marie-Pierre Chapot-Chartier, Pascal Hols

**Affiliations:** 1 Biochimie et Génétique Moléculaire Bactérienne, Institut des Sciences de la Vie, Université catholique de Louvain, Place Croix du Sud 5/L7.07.06, Louvain-la-Neuve, B-1348, Belgium; 2 INRA, UMR1319 Micalis, Jouy-en-Josas, F-78350, France; 3 AgroParisTech, UMR Micalis, Jouy-en-Josas, F-78350, France; 4 TI Food & Nutrition, Nieuwe Kanaal 9A, Wageningen, PA, 6709, The Netherlands; 5 NIZO food research, Kernhemseweg 2, Ede, ZB, 6718, The Netherlands; 6 Kluyver Centre for Genomics of Industrial Fermentation, Julianalaan 67, Delft, BC, 2628, The Netherlands; 7 Host Microbe Interactomics Group, Wageningen University, De Elst 1, Wageningen, WD, 6708, The Netherlands

**Keywords:** Lactobacillus plantarum, Peptidoglycan, Autolysin, Peptidoglycan hydrolase, Glucosaminidase, Muropeptidase

## Abstract

**Background:**

*Lactobacillus plantarum* is commonly used in industrial fermentation processes. Selected strains are also marketed as probiotics for their health beneficial effects. Although the functional role of peptidoglycan-degrading enzymes is increasingly documented to be important for a range of bacterial processes and host-microbe interactions, little is known about their functional roles in lactobacilli. This knowledge holds important potential for developing more robust strains resistant to autolysis under stress conditions as well as peptidoglycan engineering for a better understanding of the contribution of released muramyl-peptides as probiotic immunomodulators.

**Results:**

Here, we explored the functional role of the predicted peptidoglycan hydrolase (PGH) complement encoded in the genome of *L. plantarum* by systematic gene deletion. From twelve predicted PGH-encoding genes, nine could be individually inactivated and their corresponding mutant strains were characterized regarding their cell morphology, growth, and autolysis under various conditions. From this analysis, we identified two PGHs, the predicted *N*-acetylglucosaminidase Acm2 and NplC/P60 D,L-endopeptidase LytA, as key determinants in the morphology of *L. plantarum*. Acm2 was demonstrated to be required for the ultimate step of cell separation of daughter cells, whereas LytA appeared to be required for cell shape maintenance and cell-wall integrity. We also showed by autolysis experiments that both PGHs are involved in the global autolytic process with a dominant role for Acm2 in all tested conditions, identifying Acm2 as the major autolysin of *L. plantarum* WCFS1. In addition, Acm2 and the putative *N*-acetylmuramidase Lys2 were shown to play redundant roles in both cell separation and autolysis under stress conditions. Finally, the analysis of the peptidoglycan composition of Acm2- and LytA-deficient derivatives revealed their potential hydrolytic activities by the disappearance of specific cleavage products.

**Conclusion:**

In this study, we showed that two PGHs of *L. plantarum* have a predominant physiological role in a range of growth conditions. We demonstrate that the *N*-acetylglucosaminidase Acm2 is the major autolysin whereas the D,L-endopeptidase LytA is a key morphogenic determinant. In addition, both PGHs have a direct impact on PG structure by generating a higher diversity of cleavage products that could be of importance for interaction with the innate immune system.

## Background

The cell wall is an essential structure for the survival of bacteria, as it determines cell shape, and also preserves cell integrity from internal osmotic pressure [1]. In Gram-positive bacteria, peptidoglycan (PG) is a major compound of the cell wall. This polymer consists of the repeating disaccharide *N*-acetylmuramic acid-(β-1,4)-*N*-acetylglucosamine (MurNAc-GlcNAc) connected to a peptidic stem which is linked to MurNAc. The composition of the peptidic stem varies between bacteria and, in *Lactobacillus plantarum*, is composed of L-Ala, D-Glu, *meso*-diaminopimelic acid (mDAP), D-Ala and D-lactate as last moiety [2,3]. Neighboring glycan strands are cross-linked between the fourth amino-acid of the donor stem and the third amino-acid of the acceptor peptide, forming a three-dimensional network around the cell, termed sacculus [1]. Bacteria produce a variety of enzymes that are able to degrade PG. These enzymes are collectively called peptidoglycan hydrolases (PGH) or autolysins in the case they cleave PG glycan strands or cross-links of the producer strain resulting in the destruction of the PG mesh and cell lysis [1]. These enzymes have been shown to play a major role in different processes such as daughter cell separation, cell-wall turnover, autolysis, sporulation and germination, biofilm formation, resuscitation of dormant cells, and allolysis in genetic transformation [4,5]. Most of the PGHs display a modular organization composed of different domains usually associated with functions related to cell wall binding (e.g. LysM, SH3 domains) or PGH enzyme activity [6]. PGHs are divided into several major families depending on the activity of their catalytic domain; *N*-acetyl-glucosaminidases, -muramidases, and lytic transglycosylases hydrolyze β-1, 4-bonds of PG glycan strands, whereas *N*-acetyl-muramoyl-L-alanine amidases cleave the amide bond between the lactic acid side chain of MurNAc and L-Ala of the stem peptide and carboxy- and endo-peptidases cleave the peptidic stem [5]. In the last family, the D,D- and L,D-carboxypeptidases form a distinct group of PGHs since they do not destroy the PG mesh and are generally considered as PG maturation enzymes [1].

Lactobacilli, including *Lactobacillus plantarum*, are among the most predominant bacterial species of Firmicutes that are present in the mammalian intestinal tract [7]. Due to their ability to confer a health benefit on their host, specific strains of *L. plantarum* are marketed as probiotics [8,9]. It is also known that species of the genus *Lactobacillus* are able to interact with receptors of the immune system in the gastrointestinal tract through PGH-dependent processes such as autolysis and/or cell wall turnover that release muramyl-peptides or PGH fragments themselves [10-15]. In *L. plantarum* WCFS1, the analysis of its genome sequence has revealed the presence of 16 putative PGHs (carboxypeptidases are excluded), including 12 candidates displaying similarity with well-characterized PGHs, 3 hypothetical (lytic) transglycosylases containing a WY domain, and a pseudogene (*acm3*, three fragments) [12,16-18]. Among the well-characterized PGH homologs, Acm2, a putative *N*-acetylglucosaminidase composed of a catalytic domain associated to five C-terminal SH3 domains and an N-terminal *O*-glycosylated region rich in Ala, Ser, and Thr (AST domain), was previously shown to be responsible for the separation of daughter cells [19,20]. In addition, we previously demonstrated that the PGH activity of Acm2 and LytH (putative *N*-acetylmuramoyl-L-alanine amidase) could be modulated through *O*-acetylation of glycan chains of *L. plantarum* PG. MurNAc *O*-acetylation triggers LytH activity, whereas GlcNAc *O*-acetylation inhibits Acm2 activity [2].

Besides some recent studies performed in *L. rhamnosus* GG and *L. casei* BL23 that focused essentially on the characterization of specific major PGHs, information concerning the functional role of the complete arsenal of PGHs in lactobacilli is lacking [11,21,22]. In this study, we investigate the functional role of the 12 more probable candidates of the PGH complement of *L. plantarum* WCFS1 by a systematic gene deletion strategy. Characterization of mutant cell morphology, growth, and autolytic behavior, in a range of conditions showed that 4 PGHs (Acm2, Lys2, LytA, and LytH) play important roles either in the cell cycle or in the autolysis process of *L. plantarum*. Notably, we demonstrated that the putative *N*-acetylglucosaminidase Acm2 is the major autolysin of *L. plantarum* and that the putative γ-D-glutaminyl-*meso*-diaminopimelate muropeptidase LytA is a major morphogenic determinant in *L. plantarum*, thereby establishing a novel role for D,L-endopeptidases of the NLPC/P60 family.

## Methods

### Bacterial strains, plasmids, and growth conditions

The bacterial strains and plasmids used in the present study are listed in Table 1. Plasmids were constructed in *Escherichia coli* DH5α or in *Lactococcus lactis* subsp. *cremoris* NZ3900. *E. coli* was grown in LB medium with aeration at 37°C*. L. plantarum* and *L. lactis* were grown at 28°C in MRS broth (Difco Laboratories Inc., Detroit, MI) and M17 broth (BD Biosciences) supplemented with 0.5% glucose (M17-glucose), respectively. When appropriate, antibiotics were added to the media at the following concentrations: erythromycin at 250 μg/ml for *E. coli* and at 5 μg/ml for *L. plantarum* and *Lactococcus lactis*, and chloramphenicol at 10 μg/ml for *E. coli*, *L. plantarum* and *Lactococcus lactis*. Plating media were prepared by adding 2% (w/v) agar to the medium. Chemically defined medium (CDM) and stress chemically defined medium (SCDM) used in this study were prepared as previously described [23]. SCDM deviates from CDM by a two-fold higher concentration of amino acids and addition of 300 mM NaCl. *L. plantarum* was grown in SCDM at 37°C. Nisin A (Sigma-Aldrich) was routinely used at a concentration of 20 ng/ml for the induction of genes that were cloned under the control of the *nisA* promoter [24].

**Table 1:** Bacterial strains and plasmids used in this study

**Strain or plasmid**	**Characteristic(s)**	**Source or reference**
***Lactobacillus plantarum***		
NZ7100	WCFS1 *lp_0076::nisRK*	[46]
TR0010	NZ7100 *lp_2645 (acm2)::lox72*	This work
TR0011	NZ7100 *lp_3093 (lys2)::lox72*	This work
TR0012	NZ7100 *lp_1138 (acm1)::lox72*	This work
TR0013	NZ7100 *lp_1158 (lys1)::lox72*	This work
TR0014	NZ7100 *lp_1982 (lytH)::lox72*	This work
TR006	NZ7100 *lp_3421 (lytA)::lox66*-P32-*cat*-*lox71*	This work
TR0015	NZ7100 *lp_2162 (lytB)::lox72*	This work
TR0016	NZ7100 *lp_1242 (lytD)::lox72*	This work
TR0017	NZ7100 *lp_0302 (mltA)::lox72*	This work
TR0018	TR0011 *lp_2645 (acm2)::lox72*	This work
***Escherichia coli***		
DH5α	Cloning host; F^-^ ϕ80 *lacZ∆*M15 *∆(lacZYA-argF)*U169 *endA1 recA1 hsdR17 (r*_*k*_^*-*^*m*_*k*_^*+*^*) phoA supE44 thi-1 gyrA96 relA1* λ	[47]
***Lactococcus lactis***		
NZ3900	MG1363 derivative, *pepN*::*nisRK*	[30]
**Plasmids**		
pNZ5319	Cm^r^ Em^r^; pACYC184 derivative containing the *cat* gene under the control of the P32 constitutive promoter of *Lactococcus lactis* (*lox66*-P32-*cat*-*lox71* cassette)	[29]
pNZ5348	Em^r^; Cre expression vector	[29]
pNZ8048	Cm^r^; shuttle vector containing P_*nisA*_ promoter and start codon in NcoI site	[48]
pGITR001	Cm^r^ Em^r^; pNZ5319 containing both upstream and downstream homology fragments from *lp_2645 (acm2)*	This work
pGITR002	Cm^r^ Em^r^; pNZ5319 containing both upstream and downstream homology fragments from *lp_3093 (lys2)*	This work
pGITR003	Cm^r^ Em^r^; pNZ5319 containing both upstream and downstream homology fragments from *lp_1138 (acm1)*	This work
pGITR004	Cm^r^ Em^r^; pNZ5319 containing both upstream and downstream homology fragments from *lp_1158 (lys1)*	This work
pGITR005	Cm^r^ Em^r^; pNZ5319 containing both upstream and downstream homology fragments from *lp_1982 (lytH)*	This work
pGITR006	Cm^r^ Em^r^; pNZ5319 containing both upstream and downstream homology fragments from *lp_3421 (lytA)*	This work
pGITR007	Cm^r^ Em^r^; pNZ5319 containing both upstream and downstream homology fragments from *lp_2162 (lytB)*	This work
pGITR008	Cm^r^ Em^r^; pNZ5319 containing both upstream and downstream homology fragments from *lp_1242 (lytD)*	This work
pGITR009	Cm^r^ Em^r^; pNZ5319 containing both upstream and downstream homology fragments from *lp_0302 (mltA)*	This work
pGITR0010	Cm^r^; pNZ8048 derivative containing *acm2 (lp_2645)* gene in transcriptional fusion	This work
pGITR0011	Cm^r^; pNZ8048 derivative containing *lys2* (*lp_3093)* gene in transcriptional fusion	This work

Cm^r^ and Em^r^ indicate resistance to chloramphenicol and erythromycin, respectively.

### DNA techniques and electrotransformation

General molecular biology techniques were performed according to the instructions given by Sambrook *et al.*[25]. Electrotransformation of *E. coli* was performed as described by Dower *et al.*[26]. Electrocompetent *L. plantarum* and *L. lactis* cells were prepared as previously described [27]. *L. plantarum* chromosomal DNA was isolated as reported before [28]. PCR were performed with Phusion high-fidelity DNA polymerase (Finnzymes, Espoo, Finland) in a GeneAmp PCR system 2400 (Applied Biosystems, Foster City, CA). The primers used in this study were purchased from Eurogentec (Seraing, Belgium) and are listed in (Additional file 1: Table S1).

### Construction of deletion mutants

Construction of the deletion mutants of the *L. plantarum* PGH-encoding genes was performed as previously described [2,29]. Briefly, a double cross-over gene replacement strategy was used to replace the target gene(s) by a chloramphenicol resistance cassette (*lox66*-P32-*catlox71*). Subsequently, the *lox66*-P32-*catlox71* cassette was excised by temporal expression of the Cre recombinase using an unstable *cre* expression plasmid [29]. This strategy was applied for the construction of strains TR0010 (Acm2^-^), TR0011 (Lys2^-^), TR0012 (Acm1^-^), TR0013 (Lys1^-^), TR0014 (LytH^-^), TR006 (LytA^-^), TR0015 (LytB^-^), TR0016 (LytD^-^) and TR0017 (MltA^-^) (Table 1). In order to obtain the double mutant strain TR0018 (Acm2^-^ Lys2^-^), strain TR0011 (Lys2^-^) was used for a next round of mutagenesis targeting for the deletion of *acm2* following the same procedures as described above. Primers used to construct the deletion vectors and to validate the deletions events are listed in (Additional file 1: Table S1).

### Construction of the complementation vectors

The *acm2* and *lys2* ORFs were amplified by PCR using genomic DNA of *L. plantarum* NZ7100 and the primer pairs Acm2_NcoI/Acm2_XbaI or Lys2_NcoI/Lys2_XbaI, respectively (Additional file 1: Table S1). The resulting amplicons were digested with NcoI and XbaI and cloned into similarly digested pNZ8048, yielding the expression plasmids pGITR0010 and pGITR0011, respectively. These plasmids contain either *acm2* or *lys2* under the control of P_*nisA*_ that allows their induction in the presence of nisin [30]. The integrity of the two plasmids was verified by DNA sequencing. These two expression vectors were electrotransformed into *L. plantarum* TR0010 for complementation studies. Primers used to construct the complementation vectors are listed in (Additional file 1: Table S1).

### Purification and structural analysis of peptidoglycan

PG from *L. plantarum* strains was prepared as previously described [2,31]. PG was digested with mutanolysin and the resulting muropeptides were analyzed by reverse phase-high-pressure liquid chromatography (RP-HPLC) and MALDI-TOF (Matrix-Assisted Laser Desorption/Ionization - Time Of Flight) mass spectrometry as previously reported [32].

### Triton X-100-induced autolysis assays in buffer solution

*L. plantarum* strains were grown to mid-exponential phase (OD_600_=0.8). Cells were harvested by centrifugation at 5000 × *g* for 10 min at 4°C, washed once with 50 mM potassium phosphate buffer pH 7.0, and resuspended at an OD_600_ of 1.0 in 50 mM potassium phosphate buffer pH 7.0 supplemented with 0.05% Triton X-100 [2,33]. Cell suspensions were then transferred into 96-well sterile microplates with a transparent bottom (Greiner, Alphen a/d Rjin, the Netherlands) and incubated at 30°C. Autolysis was monitored by measuring the OD_600_ of the cell suspensions every 20 minutes with a Varioskan Flash multimode reader (Thermo Scientific). The extent of autolysis was expressed as the relative decrease in OD_600_ (given in percentages relative to the initial OD_600_).

### SDS-PAGE and zymogram

The cell wall hydrolyzing activity was investigated by zymogram analysis. SDS-PAGE was performed with 8% (w/v) polyacrylamide separating gels. Renaturing SDS-PAGE was performed as previously described [2,34]. The polyacrylamide gels contained *L. plantarum* NZ7100 autoclaved cells resuspended at OD_600_ of 0.8 as enzyme substrates. Disrupted cells used as samples were prepared as described before [2]. Disrupted cells were boiled in denaturing sample buffer and centrifuged 1 min at 20.000 × *g* prior to loading. After sample migration, gels were washed for 30 min in deionized H_2_O and incubated overnight at room temperature in 50 mM Tris–HCl, pH 6.8, 1 mM DTT, containing 0.1% (v/v) Triton X-100. Subsequently, the gels were washed in deionized H_2_O, followed by staining with 0.1% Methylene Blue in 0.01% (w/v) KOH, and destained in deionized H_2_O.

### Fluorescence microscopy and LIVE/DEAD staining

Microscopy analyses were performed using an Axio observer Z1 inverted microscope (Carl Zeiss). FM4-64 (Molecular Probes, Leiden, The Netherlands) and DAPI (4, 6-diamidino-2-phenylindole) (Sigma, Bornem, Belgium) staining were performed as previously reported [31]. Bacterial membrane integrity was assessed by fluorescence microscopy using a LIVE/DEAD reduced biohazard viability/cytotoxicity kit (Molecular Probes, Eugene, OR, USA) according to the manufacturer’s instructions. Analyses of micrographies were performed using the AxioVision 4.8. software (Carl Zeiss).

### Transmission and scanning electron microscopy

*L. plantarum* cells were grown overnight at 28°C in MRS broth. Bacterial pellets were washed once in PBS and fixed overnight in a phosphate buffer (0.1 M and pH 7.4) containing 2.5% glutaraldehyde for transmission electron microscopy or 4% paraformaldehyde for scanning electron microscopy. After fixation, cells were washed, postfixed with 1% osmium tetroxide for 1 h, washed again and subjected to serial dehydration with ethanol. Samples for transmission electron microscopy were embedded in resin, thin-sectioned and stained with uranyl acetate and Reynold’s lead citrate. Samples for scanning electron microscopy were prepared by critical-point drying, mounted on an aluminum stub and covered with a thin layer of gold (20–30 nm). Finally, the samples were examined using a Transmission Electron Microscope (TEM) (LEO922; Zeiss) at the Institute of Condensed Matter and Nanosciences (Université catholique de Louvain, Belgium) or a Scanning Electron Microscope (SEM) (XL-20; Philips, Eindhoven, the Netherlands) at the Unité Interfacultaire de Microscopie Electronique (Facultés Universitaires Notre-Dame de la Paix, Belgium).

## Results

### Nine of the twelve predicted peptidoglycan hydrolases could be inactivated in *L. plantarum* WCFS1

**Figure 1:**
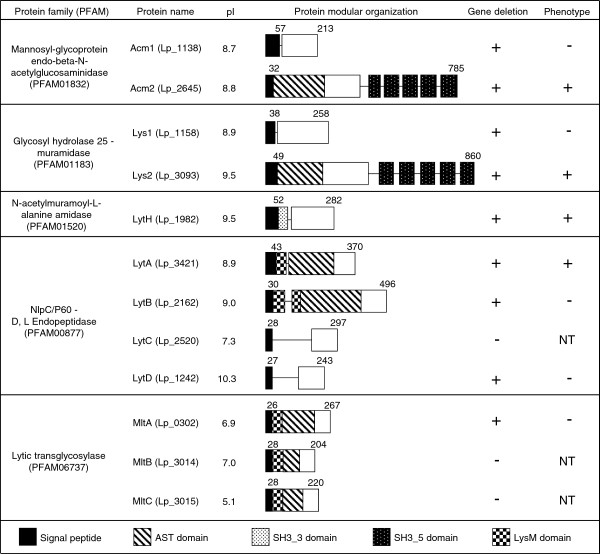
**Schematic representation of the modular organization of predicted PGHs identified in *L. plantarum *WFCS1.** Domain annotation is based on conserved domain database (CDD, http://www.ncbi.nlm.nih.gov/Structure/cdd/cdd.shtml) and PFAM database ( http://www.sanger.ac.uk/Software/Pfam/). Numbers at the beginning and at the end of each PGH indicate respectively the length (aa) of the signal peptide predicted by the SignalP 4.0 server ( http://www.cbs.dtu.dk) and of the full length protein. Isolelectric points (pI) of the predicted mature proteins are calculated by the Compute pI/Mw tool available on ExPASy ( http://web.expasy.org/compute_pi/). Cell-wall binding domains SH3_3, SH3_5, and LysM were assigned from PFAM accession numbers, PF08239, PF08460, and PF01476, respectively. The AST domain is a region of unknown function rich in alanine, serine and threonine residues shown to be *O*-glycosylated in Acm2 (Lp_2645) [19]. Symbols + and - indicate that either the gene deletion or a differential phenotype compared to the wild-type (i.e. cell morphology, growth, or autolysis) were or not obtained or observed, respectively. NT (not tested) indicates that the phenotype could not be tested since the corresponding gene deletion was not obtained.

We have previously identified 12 genes in the *L. plantarum* WCFS1 genome coding for orthologs of well-characterized PGHs of Gram-positive bacteria [12,16]. As shown in Figure 1, these putative PGHs can be classified into five families according to the PFAM accession number of their catalytic domains: Acm1 (Lp_1138) and Acm2 (Lp_2645) belong to the family of Mannosyl-glycoprotein endo-beta-*N*-acetylglucosaminidase (PF01832); Lp_1158 and Lp_3093, renamed Lys1 and Lys2, respectively, belong to the muramidase family of Glycosyl hydrolases 25 (PF01183); LytH (Lp_1982) belongs to the *N*-acetylmuramoyl-L-alanine amidase family (PF01520); Lp_3421, Lp_2162, Lp_2520, and Lp_1242, renamed LytA, LytB, LytC, and LytD, respectively, belong to the *γ*-D-Glu-mDAP muropeptidase family containing a NLPC/P60 catalytic domain (PF00877); Lp_0302, Lp_3014, and Lp_3015, renamed MltA, MltB, and MltC, respectively, belong to the lytic transglycosylase family (PF06737). All 12 PGHs possess a putative signal peptide based on SignalP 4.0 predictions ( http://www.cbs.dtu.dk/services/SignalP). Some redundancy can be observed in terms of domain organization within and between the different PGH families. Interestingly, 8 of the 12 PGHs show a modular organization where their catalytic domain is associated with at least one PG binding domain (SH3_3, SH3_5, or LysM) and/or with a domain rich in Ala, Ser and Thr (AST), recently shown to be glycosylated for *L. plantarum* WCFS1 Acm2 [19].

In order to investigate the functional role of each PGH, we aimed to construct a library of *L. plantarum* PGH mutant strains using the double cross-over gene replacement strategy based on the Cre-*loxP* system [29]. During the first step of this strategy, we attempted to inactivate the targeted open reading frames through replacement by a chloramphenicol resistance cassette (*lox66*-P32-*catlox71*). Using this approach, we obtained mutants for 9 of the 12 genes that were initially targeted (Figure 1). Despite several attempts in which over 300 integrants were tested for their genotype, we were not able to obtain double cross over events for *lytC*, *mltB*, and *mltC*, suggesting that they might be essential for bacterial survival. Subsequently, the *lox66*-P32-*catlox71* cassette was successfully excised from 8 of the 9 mutants (all except the *lytA* that could no longer be transformed by electroporation) by temporal expression of the Cre recombinase [29].

Since in our tested conditions, four PGH-deficient strains (Acm2^-^, Lys2^-^, LytA^-^, and LytH^-^) were later shown to display phenotypic differences as compared to the wild type (see below), construction of complementation vectors for each of these PGH was attempted. The cloning of *acm2* and *lys2* genes under the transcriptional control of the nisin-inducible promoter [24,30] was achieved and corresponding complemented strains were constructed. Despite numerous attempts, complementation of LytA^-^ and LytH^-^ mutant strains were not obtained since the cloning of *lytA* and *lytH* genes in various hosts (*E. coli*, *L. lactis*, and *L. plantarum*) was unsuccessful. We only obtained truncated gene copies of *lytA*, while for *lytH*, when full-length gene cloning was successful, all candidates contain point mutations resulting in inactive proteins (data not shown).

### Acm2 and LytA are involved in cell separation and cell division

**Figure 2:**
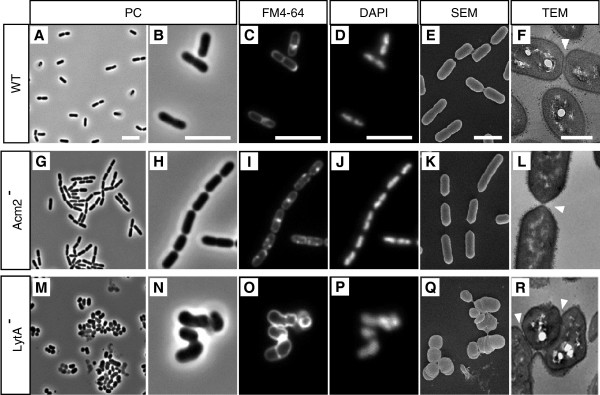
**Cell morphology of *L. plantarum *and its mutant derivatives.** NZ7100 WT (controls; **A, B, C, D, E and F**), Acm2^-^ (**G, H, I, J, K** and **L**) and LytA^-^(**M, N, O, P, Q** and **R**) mutant strains are presented. Micrographs obtained by phase-contrast (PC) optical microscopy (**A, B, G, H, M **and **N**), FM4-64 staining (**C, I** and **O**), DAPI staining (**D, J** and **P**), scanning electron microscopy (SEM) (**E, K** and **Q**) and transmission electron microscopy (TEM) (**F, L** and **R**) are shown. Cells for microscopy were grown in MRS medium at 28°C and collected in exponential growth phase. The arrows indicate the septum of dividing cells. Bar scales, 5.0 μm for PC, FM4-64 and DAPI; 5.0 nm for SEM and TEM.

As a first characterization of the 9 PGH mutant strains, their cell morphology in exponential and stationary growth phases was examined by light microscopy. Cells were grown in MRS medium, chemically defined medium (CDM), and CDM under mild stress conditions (termed SCDM medium here, see Materials and Methods). Cells were grown in CDM since a recent report by Bron *et al.*[35] showed that seven PGH-encoding genes of *L. plantarum* WCFS1 were specifically up-regulated (*acm1*, *lys2*, *lytC*, *lytD*, and *mtlB*) or down-regulated (*lytH* and *mltC*) in this growth medium under mild stress conditions such as high salt concentration and elevated growth temperature (FermDB platform, http://www.cmbi.ru.nl/fermDB and Additional file 1: Table S2). Two (*acm2*, *lytA*) out of the nine single mutants displayed a different cell morphology compared to the wild type, which was independent of the growth stage or culture conditions (data not shown and Figure 2). The *acm2* mutant is characterized by the formation of long chains of incompletely separated cells, as was previously reported for a single cross-over mutant [20] (Figure 2G and 2H). The complemented mutant carrying the *acm2* gene on a multicopy plasmid showed a complete absence of this cell chaining phenotype (data not shown). Concerning the *lytA* mutant, it is characterized by the loss of its rod-shape, the presence of a range of different cell morphotypes, and a tendency to form clumps of aggregated cells (Figure 2M and 2N). To investigate if *acm2* and *lytA* mutant cells displayed additional unusual features such as abnormal separation of nucleoids or multiple septa, DNA and cell membrane were stained with DAPI and FM4-64, respectively. For the *acm2* mutant, cells displayed well-separated nucleoids and no multiple septa could be observed, thus representing a phenotype very similar to wild-type cells (Figure 2C, 2D, 2I, and 2J). For the *lytA* mutant, despite its aberrant cell morphology, all cells retained DNA material and individual cells did not show multiple septation events (Figure 2N, 2O and 2P). In order to further investigate the cell structure of the mutant collection in more detail, transmission and scanning electron microscopy (TEM and SEM) were performed on cells grown in MRS conditions. Only the *acm2* and *lytA* mutants showed cell structure defects compared to the wild type (data not shown and Figure 2). For these two mutants, differences were observed in the septal area at the separation stage of daughter cells. While newly formed poles of wild-type cells could be qualified as blunt (Figure 2E, Additional file 1: Figure S1A), they appeared to be more pointed in *acm2* mutant cells, probably resulting from a delayed or incomplete cell separation (Figure 2K and 2L; Additional file 1: Figure S1A). Concerning *lytA* mutant cells, septa are frequently misplaced and showed a range of abnormalities with accumulation of cell wall material (Figure 2Q and 2R; Additional file 1: Figure S1B) compared to wild-type cells which contain well-defined septa located at mid-cell (Figure 2F; Additional file 1: Figure S1B). These aberrant septation events explained the range of cell morphotypes and the cell aggregation phenotype of the *lytA* mutant.

These morphological observations demonstrate that Acm2 and LytA are key actors of the cell cycle within the PGH complement of *L. plantarum*, since they play an important role during late cell separation and division process, respectively.

### LytA inactivation has a strong negative impact on growth and viability

**Figure 3:**
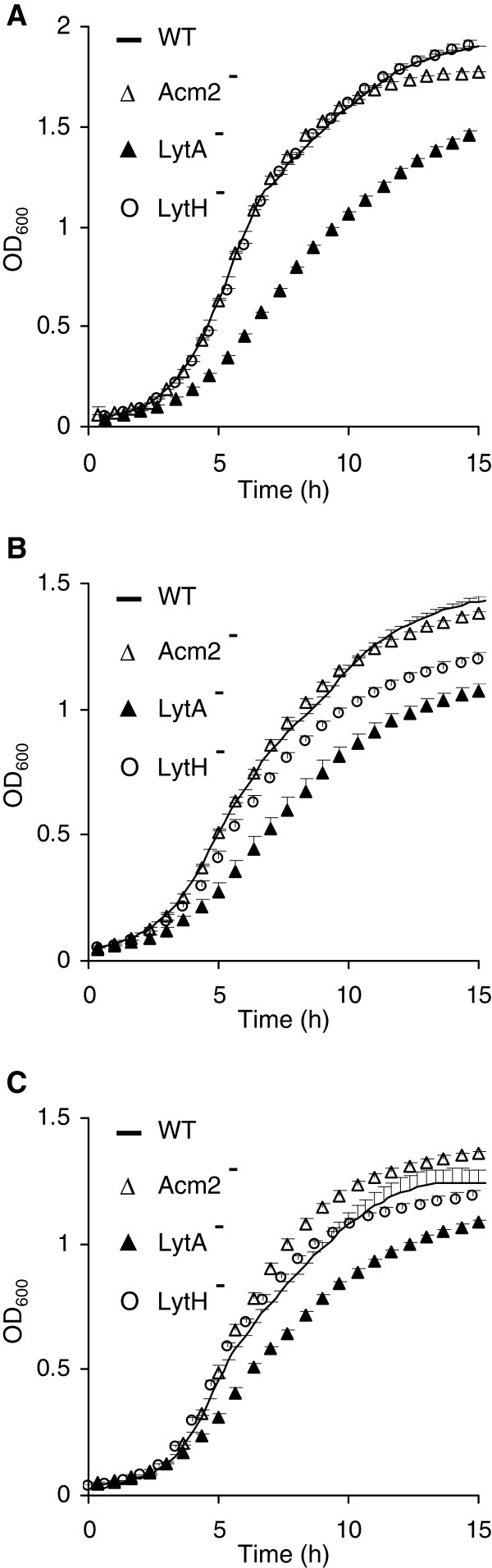
**Growth curves of *L. plantarum *and its mutant derivatives.** NZ7100 (WT, lines), *acm2* mutant (Acm2^-^, open triangles), *lytA* mutant (LytA^*-*^, black triangles) and *lytH* mutant (LytH^-^, open circles) grown in MRS (**A**), CDM (**B**) and SCDM (**C**) are presented. Mean values ± standard deviations (n=3). OD_600_ values of the *lytA* mutant (MRS, CDM, and SCDM) and the *lytH* mutant (CDM) are all significantly different from the WT between 3 and 15 h. Significance is based on Student’s *t* test with a *P* value of <0.001.

To further characterize our collection of PGH mutant strains, their growth characteristics were compared to the wild type in different conditions (MRS, CDM and SCDM). As shown in Figure 3, the *acm2* mutant displayed a similar growth curve compared to the wild type, whereas the *lytA* mutant appeared strongly affected in all tested conditions: it displayed a lower growth rate during exponential phase and a reduced OD_600_ after 15 h of incubation. No significant differences were observed for the other PGH mutant strains under these three different growth conditions (Additional file 1: Figure S2), except for the *lytH* mutant that showed a growth delay only when grown in CDM, albeit to a lesser extent as compared to the *lytA* mutant (Figure 3B). Taken together, these results show that the *acm2* mutant does not exhibit major growth differences compared to the wild type, whereas the *lytA* mutant has a strong and constant growth defect that could result from a loss of viability. To test this hypothesis, we performed LIVE/DEAD viability assays based on cell membrane integrity for both mutant cells collected in exponential and stationary growth phases from MRS cultures. Our results showed that the *lytA* mutant displays a higher percentage of injured cells compared to the wild type in both exponential and stationary growth phases (19% vs. 0.3% and 26.5% vs. 4%, respectively) whereas the *acm2* mutant shows a lower percentage of dead cells than the wild type (0.25 vs. 0.3% and 0.75% vs. 4%, respectively) (Additional file 1: Figure S3).

These results underpinned the prominent role of LytA in *L. plantarum* cell integrity that is apparent in a range of growth conditions, but also revealed that LytH is important under specific conditions (CDM).

### Acm2 is the major autolysin of *L. plantarum*

**Figure 4:**
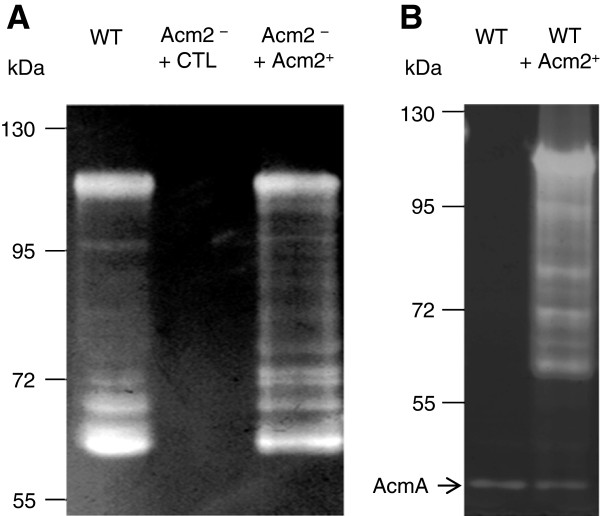
**Zymogram with various cell extracts against dead cells of *L. plantarum *WT.** (**A**) Cell extracts were prepared from L. plantarum NZ7100 (WT), the acm2 mutant carrying the empty plasmid pNZ8048 (Acm2^–^ + CTL) and the *acm2* mutant overexpressing *acm2* (Acm2^–^ + Acm2^+^) grown in the presence of the nisin inducer in MRS. (**B**) Cell extracts were prepared from *L. lactis* NZ3900 (WT) and *L. lactis* NZ3900 overexpressing *acm2* (WT + Acm2^+^) grown in the presence of the nisin inducer in M17-glucose. The arrow indicates the activity of AcmA of *L. lactis.*

In order to visualize the autolytic content of the different PGH mutant strains, zymography experiments were performed. Total cell extracts from cells grown in MRS, CDM, or SCDM, were separated by SDS-PAGE in the presence of *L. plantarum* NZ7100 autoclaved cells as substrate. An identical banding pattern of hydrolytic activity was observed in all samples except for the *acm2* mutant where no activity could be detected in any of the growth condition applied (illustrated for cells grown in MRS in Figure 4 and Additional file 1: Figure S4; and data not shown). Since it has been previously shown that the isoelectric point (pI) of PGHs is important for zymographic assay [36], ranging from 5.1 to 10.3 for *L. plantarum* PGHs (Figure 1), zymographic analysis was performed with cell extracts of the *acm2* mutant at different pH (between 4.7 and 8.8). However, no additional activity could be detected (data not shown). Dead cells of *Micrococcus lysodeikticus*, a sensitive substrate classically used for zymographic assays, was also used, resulting in the same outcome (data not shown). To confirm that all the bands of activity positioned between 110 and 60 kDa could be attributed to Acm2 and its breakdown products, zymographic assays were preformed with the complemented mutant strain of *L. plantarum* and a *L. lactis* strain overproducing Acm2 from the same multicopy plasmid (Figure 4). Interestingly, a similar banding pattern was observed for *L. plantarum* and *Lactococcus lactis*, showing that all the bands of activity results from Acm2 and its degradation products. These observations are a first indication that Acm2 acts as the major PG hydrolytic enzyme of *L. plantarum*.

**Figure 5:**
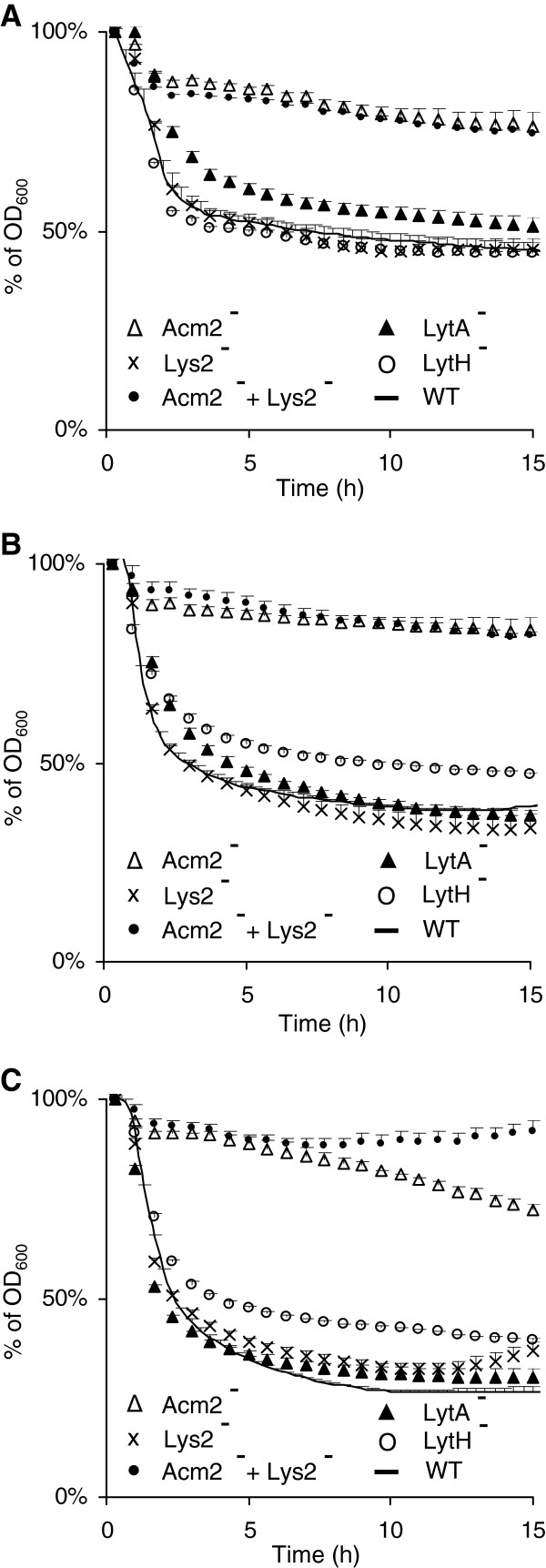
**Autolysis of *L. plantarum *and its mutant derivatives in presence of Triton X-100 (0.05%).** Wild type (WT) is represented by a line, *acm2* mutant (Acm2^–^) by open triangles, *lys2* mutant (Lys2^–^) by crosses, *lytA* mutant (LytA^–^) by black triangles, *lytH* mutant (LytH^-^) by open circles, and double *acm2 lys2* mutant (Acm2^–^ + Lys2^–^) by black circles. Cells were grown in MRS (**A**), CDM (**B**) and SCDM (**C**). Mean values ± standard deviations (n=3). OD_600_ values of the *acm2* mutant (MRS, CDM, and SCDM), *lys2* mutant (SCDM), *lytA* mutant (MRS), *lytH* mutant (CDM and SCDM) and double *acm2 lys2* mutant (MRS, CDM, and SCDM) are all significantly different from the WT between 4 and 15 h. OD_600_ values of the *acm2 lys2* mutant (SCDM) are all significantly different from the *acm2* mutant (SCDM) between 12 and 15 h. Significance is based on Student’s *t* test with a *P* value of <0.001.

To better characterize the autolytic behavior of the different PGH mutant strains, Triton X-100-induced autolysis assays were initially performed with cells grown in MRS medium (OD_600_ of 0.8). As shown in Figure 5A, the *acm2* and *lytA* mutants exhibited a higher autolysis resistance compared to the wild type, whereas no differences in terms of autolysis were observed for the other PGH-deficient strains (illustrated for the *lys2* and *lytH* mutants in Figure 5A; and Additional file 1: Figure S5). When autolysis assays were performed with cells grown in CDM or SCDM, differences in terms of autolysis resistance appeared (Figure 5B and 5C). Under these growth conditions, *acm2* deficiency still conferred the higher resistance to autolysis, but interestingly, *lytH* deficiency (both CDM and SCDM) and *lys2* deficiency (only SCDM) also resulted in a reduced autolysis. Considering the facts that *lys2* displays a modular organization very similar to Acm2 and that both enzymes are potential hydrolases of glycan strands (Figure 1), we hypothesized that they could act complementary in autolysis resistance. In order to test this hypothesis, a double *acm2 lys2* mutant was constructed and evaluated for its autolytic behavior. In the case of SCDM growth conditions, the double mutant displayed a higher autolysis resistance compared to the single *acm2* mutant during an incubation period of 7 to 15 hours (Figure 5C). These results corroborate the transcriptomic data that revealed that *lys2* was specifically induced in SCDM compared to CDM, whereas the expression profile of *acm2* remained unchanged (Additional file 1: Table S2). These observations support the hypothesis of a complementary role of these two PGHs. In contrast, in the same stressful growth conditions, the *lytA* mutant displayed a decrease of its autolysis resistance compared to MRS conditions and behaved as the wild type (Figure 5A and 5C). The absence of impact of LytA inactivation regarding to autolysis in SCDM may be explained by the induction of *lytC* and *lytD* that could complement *lytA* inactivation (Additional file 1: Table S2). No differences of autolysis resistance were observed for cells grown in CDM or SCDM for the other PGH-deficient strains (Additional file 1: Figure S5).

Taken together, these data show that Acm2 is the dominant autolysin of *L. plantarum* but also that Lys2, LytH and LytA can contribute to autolysis under specific growth conditions. These results are in agreement with DNA microarray expression profiles that have shown that growth conditions and more particularly mild stress conditions can modulate the expression of PGH-encoding genes [35].

### Lys2 and Acm2 are redundant peptidoglycan hydrolases involved in cell separation

**Figure 6:**
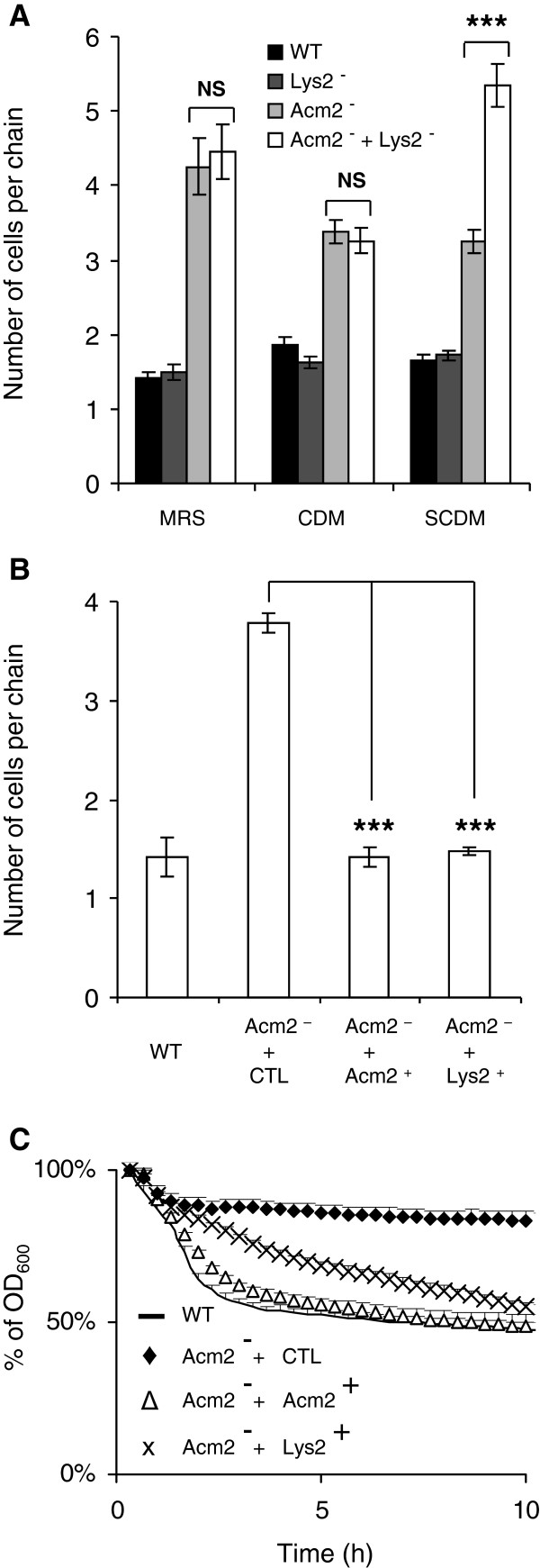
**Study of the redundancy between Acm2 and Lys2 for cell chaining and autolysis phenotypes.** (**A**) Number of cells per chain in *L. plantarum* NZ7100 (WT), *acm2* mutant (Acm2^–^), *lys2* mutant (Lys2^–^), and double *acm2 lys2* mutant (Acm2^–^ + Lys2^–^) in different growth conditions (MRS, CDM and SCDM). (**B**) Number of cells per chain in *L. plantarum* NZ7100 (WT), *acm2* mutant carrying the empty plasmid pNZ8048 (Acm2^–^ + CTL), and *acm2* mutant overexpressing *acm2* (Acm2^–^ + Acm2^+^) or *lys2* (Acm2^–^ + Lys2^+^) grown in presence of the nisin inducer in MRS. (**C**) Autolysis of *L. plantarum* and its mutant derivatives in presence of Triton X-100 (0.05%). Wild type is represented by a line (WT), *acm2* mutant carrying the empty plasmid pNZ8048 (Acm2^–^ + CTL) by black diamonds, and *acm2* mutant overexpressing *acm2* (Acm2^–^ + Acm2^+^) and *lys2* (Acm2^–^ + Lys2^+^) by white triangles and crosses, respectively. For panels A and B, mean values ± standard deviations (n=3); significance based on Student’s *t* test; ***, *P* value of <0.001. NS, not significantly different. For panel C, mean values of three independent experiments ± standard deviations (with 3 repetitions for each). OD_600_ values of the wild type, *acm2* mutant overexpressing *acm2* (Acm2^–^ + Acm2^+^), and *acm2* mutant overexpressing *lys2* (Acm2^–^ + Lys2^+^) are all significantly different from the *acm2* mutant carrying the empty plasmid pNZ8048 (Acm2^–^ + CTL) between 2 and 10 h. Significance is based on Student’s *t* test with a *P* value of <0.001.

To further support the hypothesis of a functional redundancy between *lys2* and *Acm2*, additional investigations of the phenotypes of the corresponding mutants were performed. Since the *acm2* mutant cells have a phenotype that includes a defect in cell separation, we compared the number of cells per chain exhibited by the *acm2*, *lys2* single mutants and the double mutant with the wild type strain grown in MRS, CDM, or SCDM (Figure 6A). While the inactivation of Acm2 was mainly responsible for the chaining phenotype in all conditions tested, our data also revealed that the number of cells per chain of the double *acm2 lys2* mutant was significantly increased compared to the single *acm2* mutant when grown in SCDM. This observation indicates that Lys2 contributes to Acm2-driven cell separation of daughter cells only under these growth conditions. To support these data, the *acm2* mutant complemented either by *acm2* or *lys2* were compared. The functionality of Lys2 in the complemented strain was validated by zymogram analysis (Additional file 1: Figure S6). Both Acm2 and Lys2 were able to fully complement the phenotype of the *acm2* mutant regarding to the cell separation defect (Figure 6B), whereas Lys2 was slightly less efficient than Acm2 to restore Triton X-100 induced autolysis (Figure 6C). These results strongly suggest that Acm2 and Lys2 could fulfill similar physiological roles despite their different predicted activities.

### Analysis of the peptidoglycan composition of Acm2 and LytA-deficient strains reveals their hydrolytic activity

**Figure 7:**
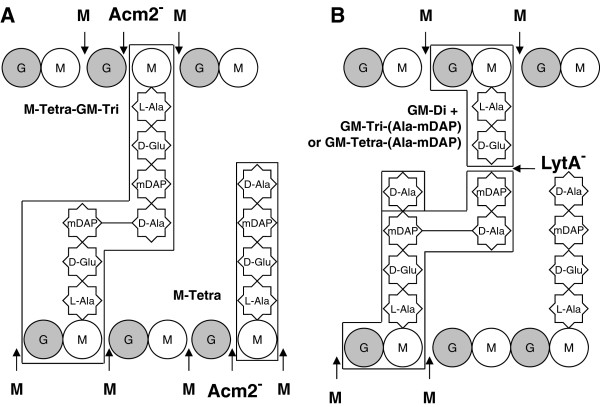
***L. plantarum *PG structure and modification of muropeptide content resulting from PGH inactivation.** Muropeptides that disappear or are strongly reduced resulting from the inactivation of either Acm2 (**A**) or LytA (**B**) are boxed. M, MurNAc; G, GlcNAc; Disaccharide, GM. M-Tetra, MurNAc-tetrapeptide; M-Tetra-GM-Tri, MurNAc-tetrapeptide crosslinked to a disaccharide-tripeptide; GM-di, disaccharide-dipeptide; GM-Tri-(Ala-mDAP), disaccharide-tripeptide crosslinked to the dipeptide L-Ala-mDAP from a second peptide stem; GM-Tetra-(Ala-mDAP), disaccharide-tetrapeptide crosslinked to the dipeptide L-Ala-mDAP from a second peptide stem. Cleavage sites of PGHs are indicated by arrows: M (boldface), mutanolysin (*N*-acetylmuramidase); Acm2^-^, absence of cleavage by a *N*-acetylglucosaminidase in the PG of the Acm2-deficient strain; and LytA^-^, absence of cleavage by a *γ*-D-Glu-mDAP muropeptidase in the PG of the LytA-deficient strain.

From our phenotypical analyses, Acm2 and LytA were concluded to be the two major PGHs of *L. plantarum* based on their importance for cell morphology and autolysis. Therefore, we investigate the composition of the PG of their respective mutant strains in order to determine whether they lead to any PG remodeling effect and to obtain a first indication of their enzymatic activity. To this end, we extracted PG from both mutants and digested it with the *N*-acetylmuramidase mutanolysin. The resulting muropeptides were separated by RP-HPLC and their elution profile was compared to the wild type for which a detailed analysis of the PG composition was recently described by Bernard *et al.*[2]. Noteworthy, the wild-type muropeptide profile contains a number of peaks that are likely products of PG digestion by endogenous PGHs [2]. In the Acm2 deficient strain, we observed the complete disappearance of a peak corresponding to a MurNAc linked to a tetrapeptide (M-Tetra) and a strong reduction of a peak identified as a dimer corresponding to a disaccharide-tetrapeptide-MurNAc-tripeptide (G-M-Tri-M-Tetra) or MurNAc-tetrapeptide-disaccharide-tripeptide (M-Tri-GM-Tetra) (Figure 7A and see peaks n°3 and 23c in Additional file 1: Table S3). These two muropeptides are deprived of one GlcNAc residue and result from *N*-acetylglucosaminidase activity. In the LytA deficient strain, 8 peaks were either absent or strongly reduced. All these peaks correspond to muropeptides resulting from a cleavage of the peptide stem between D-Glu and mDAP in the wild type by an enzyme with a *γ*-D-Glu-mDAP-muropeptidase specificity (Figure 7B and see peaks n°5, 8, 15, 18, 20, 21, 25, and 27b in Additional file 1: Table S3).

These results pinpoint that the presence of Acm2 and LytA have a direct impact on PG structure with a more pronounced effect for LytA, which generated a higher diversity of cleavage products. These data also suggest that Acm2 cleaves between GlcNAc and MurNAc as expected from its predicted *N*-acetylglucosaminidase activity and its inhibition by *O*-acetylation of GlcNAc [2]. Concerning LytA, our data indicate that it cleaves PG between D-Glu and mDAP, suggesting that LytA displays a *γ*-D-Glu-mDAP muropeptidase activity.

## Discussion

The PGH complement of *L. plantarum* WCFS1 contains at least 12 putative PGHs that were all predicted to be secreted. Moreover, most of them are anticipated to display a modular organization (8 PGHs), composed of catalytic domains linked to PG binding domains (SH3 or LysM). Furthermore, we highlighted that more than half of the PGHs of *L. plantarum* harbored a domain rich in Ala, Ser and Thr (named AST) which was recently shown to be glycosylated [19]. The predominance of this domain among *L. plantarum* PGHs suggests that it may play a general role in the control of activity and/or stability of these enzymes, as was recently also hypothesized for a glycosylated PGH of *L. rhamnosus* GG [21]. However, the functional role of the AST domain and of its glycosylated state in *L. plantarum* regarding to PGH activity/stability but also for spatial PGH localization remains to be investigated. In addition, it was recently shown that a similar domain rich in Ser and Thr (STp) from an extracellular protein of *L. plantarum* was resistant to intestinal proteolysis and was able to modulate in vitro cytokine production from intestinal dendritic cells [37]. This reveals that such domain could be of importance in the dialogue between intestinal bacteria and the immune system.

In order to elucidate the physiological function(s) of *L. plantarum* PGHs, we underwent a systematic gene deletion strategy to obtain stable, marker-free mutants for each of them. As a result, 9 PGHs were identified as permissive for inactivation, whereas 3 PGHs remained refractory to deletion despite various attempts, suggesting that they may play an essential role in *L. plantarum*. Among these three PGHs, two belongs to the lytic transglycosylase family whose functional role is poorly understood in Gram-positive bacteria [38]. We next investigated the role of the 9 PGHs with respect to cell morphology, growth, and autolysis in various conditions and identified four PGHs, namely Acm2, LytA, Lys2, and LytH, that could be associated to one or more phenotypic defect(s). Among these, Acm2 and LytA caught our attention since their deficiency resulted in the most severe morphological phenotypes. Acm2, is probably a *N*-acetylglucosaminidase, based on the analysis of the PG structure of its respective mutant strain. Preliminary results of PG degradation experiments with purified Acm2 support the proposed *N*-acetylglucosaminidase activity (T. Rolain, unpublished data). Phase-contrast, TEM and SEM microscopy show that Acm2 is strictly involved in the last step of cell separation during the septation process. This result is consistent with previous observations of an *acm2* single cross-over mutant and the functional role of AcmA, the major *N*-acetylglucosaminidase of *Lactococcus lactis*, which shares 34% of identity with Acm2 and is also dedicated to cell separation in this species [20,39]. For the second enzyme, renamed LytA, we provided strong evidence for its classification as a *γ*-D-Glu-mDAP muropeptidase based on the PG analysis of the mutant strain. The proposed LytA endopeptidase activity is consistent with the fact that all the previous known members of the NlpC/P60 family were characterized as *γ*-D-Glu-diaminoacid endopeptidases [11,22,40-42]. Unfortunately, all attempts to clone the corresponding gene or to purify its product in order to definitively prove its enzymatic activity remain so far unsuccessful. The absence of LytA results in a strongly hampered growth, loss of viability, and severe structural defects of the cell wall. However, we cannot exclude that polar effects on flanking genes might contribute to the observed phenotype, since we were unable to complement this mutant. Notably, the *lytA* gene is predicted to be monocistronic and flanked by genes which are completely unrelated to morphogenesis or cell wall assembly. Interestingly, suppressor mutants were obtained at low frequency without any genetic reorganization of the disrupted locus but displaying a wild-type cell morphology (data not shown), that may suggest that a gene encoding another D,L-endopeptidase of the NlpC/P60 family is activated to counteract the effects of *lytA* inactivation. Furthermore, numerous attempts to construct a double LytA/LytB-deficient strain remained so far unsuccessful, suggesting that both enzymes may play redundant but essential roles in *L. plantarum*. To our knowledge, this is the first time that the inactivation of a PGH containing an NlpC/P60 catalytic domain displays such a severe phenotype while the inactivation of members of this family, including members recently characterized in lactobacilli, leads to a cell-chaining phenotype similar to Acm2 inactivation [6,11,22]. Interestingly, deficiencies in the closely related PG endopeptidases belonging to the cysteine, histidine-dependent amido-hydrolases/peptidase (CHAP) family, such as PcsB from *Streptococcus pneumoniae* and LytN from *Staphylococcus aureus*, also strongly affect growth, cell morphology, and cell wall structure [43,44]. Our data suggest that NlpC/P60 endopeptidases can also be implicated in other physiological roles than daughter cell separation and probably play an important role during the assembly of the cell wall by cooperating with the PG synthesis machinery during the division process, as recently shown for the CHAP endopeptidase PcsB from *S. pneumoniae*[45]. Based on a recent transcriptome profiling study showing that the expression of a large set of PGH-encoding genes from *L. plantarum* are modulated by mild stress conditions, such as high salt concentration and elevated growth temperature [35], the contribution of each PGH to growth and autolysis was examined in different growth media as well as under stress conditions. These experiments revealed that Acm2 was responsible for the major part of the autolytic activity in all growth conditions tested and should be considered as the major autolysin of *L. plantarum* WCFS1. In addition, these experiments also showed that Lys2, LytH, and LytA contribute to the autolytic process but to a lesser extent than observed for Acm2 and only under specific growth conditions. We also show that the autolysis of Acm2- and LytA-deficient strains is increased under mild stress conditions showing that stress alters the composition of the PGH complement in *L. plantarum*, corroborating previously obtained transcriptome data [35]. We hypothesized that Acm2 and Lys2, as well as LytA and other members of the NlpC/P60 family, displayed functional redundancy in specific growth conditions. This was further investigated for the potential redundancy between the *N*-acetylglucosaminidase Acm2 and the putative *N*-acetylmuramidase Lys2 where we show that Lys2 could fulfill the functional role of Acm2 regarding to autolysis and cell separation of daughter cells.

## Conclusions

In conclusion, we show that the *N*-acetylglucosaminidase Acm2 and the *γ*-D-Glu-mDAP muropeptidase LytA are pivotal in the physiology of *L. plantarum*. Acm2 is the major autolysin under all conditions tested and is functionally involved in the final step of cell separation during division while LytA is playing a major morphogenic role since its absence not only resulted in delayed growth and loss of viability but also led to severe structural defects of the cell wall. For future applications, the identification of Acm2, in association with Lys2 under stress conditions, as key players in autolysis resistance offers the possibility to develop more robust starter strains or probiotics. In addition, the capacity to modulate *L. plantarum* PG composition by PGH inactivation could change its immunomodulatory properties by impacting on the repertoire of released muramyl peptides interacting with receptors of the innate immune system.

## Abbreviations

AST: Domain rich in Ala, Ser, and Thr; CDM: Chemically define medium; CHAP: Cysteine, histidine-dependent amido-hydrolases/peptidase; GlcNAc: *N*-acetylglucosamine; mDAP: *meso*-diaminopimelic acid; MurNAc: *N*-acetylmuramic acid; PG: Peptidoglycan; PGH: Peptidoglycan hydrolase; SCDM: stress chemically define medium; SDS-PAGE: SDS polyacrylamide gel electrophoresis; SEM: Scanning electron microscopy; TEM: Transmission electron microscopy; WT: Wild type.

## Competing interests

The authors declare that they have no competing interests.

## Authors’ contribution

PH and TR designed the project; TR, PC, and EB performed the experimental work; TR, EB, PC, PAB, MK, MPCC and PH analysed the data; TR and PH wrote the paper; PAB, MK, EB, and MPCC critically reviewed the paper. All authors approved the final manuscript.

## Supplementary Material

Additional file 1 **Table S1.** Primers used for cloning and validation. **Table S2.** Transcriptomic data of PGH-encoding genes of *L. plantarum* WCFS1 grown in chemically defined medium (CDM) at 28°C (fermentors F12 and F27, mean value) vs. CDM + 300mM NaCl at 28°C (fermentors F6 and F28, mean value), CDM at 37°C (fermentor F18), and CDM + 300mM Nacl + two-fold higher concentration of amino-acids at 37°C (defined here as SCDM conditions) (fermentor F21) (FermDB platform, http://www.cmbi.ru.nl/fermDB, [1]). **Table S3**. Differences in muropeptide composition resulting from *N*-acetylglucosaminidase and γ-D-Glu-mDAP muropeptidase activities between *L. plantarum* NZ7100 (WT) and TR0010 (Acm2–) or TR006 (LytA–). **Figure S1.** Cell separation defects of Acm2- and LytA- mutant strains compared to NZ7100 (WT, control). A. Micrographs obtained by scanning electron microscopy (SEM) showing more pointed poles (white arrows) after cell separation for Acm2- mutant cells compared to WT cells. B. Transmission electron microscopy (TEM) showing altered septum formation (white arrows) for LytA- mutant cells (IV, V, VI, VII, VIII, and IX) compared to WT cells (I, II, and III). Growth conditions as defined in the legend of Figure 2. **Figure S2.** Growth curves of *L. plantarum* NZ7100 (WT), *acm1* mutant (Acm1-), *lys1* mutant (Lys1-), *lys2* mutant (Lys2-), *lytB* mutant (LytB-), *lytD* mutant (LytD-), *mtlA* mutant (MtlA-), in MRS (A), CDM (B) and SCDM (C). Mean values (n=3). **Figure S3.** Effect of *acm2* and *lytA* mutations on cell integrity. Percentage of dead cells was monitored in MRS medium in the exponential (6 h) and stationary (16 h) growth phases for *L. plantarum* NZ7100 (WT), Acm2–, and LytA–. Cell counting was performed by epifluorescence microscopy using propidium iodide (red; labels damaged cells) and SYTO-9 (green; labels all cells). The percentage of damaged cells labeled with propidium iodide was calculated with respect to the total number of cells labeled with SYTO-9. Enumeration was done for a minimum of 300 cells for each strain. **Figure S4.** Zymogram with cell extracts of *L. plantarum* NZ7100 (WT), *acm2* mutant (Acm2–), *lys2* mutant (Lys2–), *acm1* mutant (Acm1–), *lys1* mutant (Lys1–), *lytH* mutant (LytH–), *lytA* mutant (LytA–), *lytB* mutant (LytB–), *lytD* mutant (LytD–) and *mltA* mutant (MltA–) against dead cells of WT. **Figure S5.** Autolysis curves in presence of Triton X-100 (0.05%) of *L. plantarum* NZ7100 (WT), *acm1* mutant (Acm1-), *lys1* mutant (Lys1-), *lytB* mutant (LytB-), *lytD* mutant (LytD-), *mtlA* mutant (MtlA-), in MRS (A), CDM (B) and SCDM (C). Mean values (n=3). **Figure S6.** Zymogram with cell extracts of *L. plantarum* NZ7100 (WT), *acm2* mutant carrying the empty plasmid pNZ8048 (Acm2– + CTL), and *acm2* mutant overexpressing *lys2* (Acm2– + Lys2+) grown in presence of the nisin inducer in MRS against dead cells of WT. (PDF 716 kb) Click here for file
