# Supplementary material for: Identification of key peptidoglycan hydrolases for morphogenesis, autolysis, and peptidoglycan composition of Lactobacillus plantarum WCFS1
Source: Microb Cell Fact. 2012 Oct 15;11:137. doi: 10.1186/1475-2859-11-137 (PMC3533731; doi:10.1186/1475-2859-11-137)
Supplement: Additional file 1 — Table S1. Primers used for cloning and validation. Table S2. Transcriptomic data of PGH-encoding genes of L. plantarum WCFS1 grown in chemically defined medium (CDM) at 28°C (fermentors F12 and F27, mean value) vs. CDM + 300mM NaCl at 28°C (fermentors F6 and F28, mean value), CDM at 37°C (fermentor F18), and CDM + 300mM Nacl + two-fold higher concentration of amino-acids at 37°C (defined here as SCDM conditions) (fermentor F21) (FermDB platform, http://www.cmbi.ru.nl/fermDB, [1]). Table S3. Differences in muropeptide composition resulting from N-acetylglucosaminidase and γ-D-Glu-mDAP muropeptidase activities between L. plantarum NZ7100 (WT) and TR0010 (Acm2–) or TR006 (LytA–). Figure S1. Cell separation defects of Acm2- and LytA- mutant strains compared to NZ7100 (WT, control). A. Micrographs obtained by scanning electron microscopy (SEM) showing more pointed poles (white arrows) after cell separation for Acm2- mutant cells compared to WT cells. B. Transmission electron microscopy (TEM) showing altered septum formation (white arrows) for LytA- mutant cells (IV, V, VI, VII, VIII, and IX) compared to WT cells (I, II, and III). Growth conditions as defined in the legend of Figure 2. Figure S2. Growth curves of L. plantarum NZ7100 (WT), acm1 mutant (Acm1-), lys1 mutant (Lys1-), lys2 mutant (Lys2-), lytB mutant (LytB-), lytD mutant (LytD-), mtlA mutant (MtlA-), in MRS (A), CDM (B) and SCDM (C). Mean values (n=3). Figure S3. Effect of acm2 and lytA mutations on cell integrity. Percentage of dead cells was monitored in MRS medium in the exponential (6 h) and stationary (16 h) growth phases for L. plantarum NZ7100 (WT), Acm2–, and LytA–. Cell counting was performed by epifluorescence microscopy using propidium iodide (red; labels damaged cells) and SYTO-9 (green; labels all cells). The percentage of damaged cells labeled with propidium iodide was calculated with respect to the total number of cells labeled with SYTO-9. Enumeration was done for a minimum of 300 cells for eac [file 1475-2859-11-137-S1.pdf]

Table S1. Primers used for cloning and validation

Primer name	Sequence[a]	Mutant strain or plasmid
**Primers used for construction of deletion vectors**		
Acm2_1	5’-AATAGCTTTTTGTTGTTCAGGTG-3’	TR0010
Acm2_2_II	5’-TGGCTCCGATATCTGAGTCGC -3’	
Acm2_3_II	5’-ACTTAGCAATAATGAAGTTACTAC -3’	
Acm2_4	5’-ATTCAGGAATCGTATCACATTGTGC-3’	
Lys2_1	5’-TTGTCGAAGAAGCTGGTGCTCC -3’	TR0011
Lys2_2_bis	5’-AACTCTGGTGCTTACCATTACG-3’	
Lys2_3	5’-AAGGGGATTTAGTATAATAATTG -3’	
Lys2_4	5’-TACCGAAATGAATTTGATGCC-3’	
Acm1_1	5’-AACGGTAAAGTAACCATTCTCATACG-3’	TR0012
Acm1_2	5’-ATCATAGCCGCCTCCTTTCTGG -3’	
Acm1_3_bis	5’-TATGCGACTGATCCAAGTTATGC-3’	
Acm1_4	5’-TTCTAGTTCTAGCCGCTCACC-3’	
Lys1_1	5’-AAGCATTTTACAGAGATTACGG-3’	TR0013
Lys1_2	5’-AATCCTTTGCCTACAGCATTAAAGC-3’	
Lys1_3	5’-AACGGGACTCGTCAAGAGTTC-3’	
Lys1_4	5’-AACAGTTTATTAGCTGCTAGGCTG-3’	
LytH_1	5’-ACCATCGTGGCATCAATCTCG-3’	TR0014
LytH_2	5’-TGCTTTGTCACCAACTAGATTTAAG-3’	
LytH_3	5’-AAGTACCCTCAAGAAGTAGC-3’	
LytH_4	5’-TGTGATAACTGATGATTGTAGC-3’	
LytA_1	5’-TACGATTGGCTCCTCTGAAGC-3’	TR006
LytA_2	5’-ATTAAGAGCACAGCTTACTACAC-3’	
LytA_3	5’-AAAAAGAATGTCACGTATGTAC-3’	
LytA_4	5’-AATACACTTTTAGTATACCGC-3’	
LytB_1	5’-ATGAAACCTTGAAAAACGAACACC-3’	TR0015
LytB_2	5’-ATGCCAAGTTTTGCAGGTACG-3’	
LytB_3	5’-TAGGACCTTGCCCGTTGTATGTGC-3’	
LytB_4	5’-TTGGCGTGTGGAAAGAGTGTTCG-3’	
LytD_1	5’-AAACTGGCAAAACGGTATCACC-3’	TR0016
LytD_2	5’-ATGATTGACGCTCAGGACAATGG-3’	
LytD_3	5’-AACAATCAGTGTGATAAGACG-3’	
LytD_4	5’-TACGGTGTATACCTTTCATATCC-3’	
MltA_1	5’-TTGATGAAGTTGCTCGTCATTGG-3’	TR0017
MltA_2	5’-AACGCACAAGGCATGGCTGC-3’	
MltA_3	5’-AGCGAATAAAGCTAATGCAGC-3’	
MltA_4	5’-ATCGGTAAGTACCAATTAAGTGC-3’	
**Primers used for the validation of deletions**		
Check_Acm2_UP	5’-AAGTGTTGACGCCGCTTAACC-3’	TR0010
Check_Acm2_DO	5’-TTAGCAATGTTTGTAGCACGGGC-3’	
Check_Lys2_UP	5’-AAGCACGCCTCTAATACACC-3’	TR0011
Check_Lys2_DO	5’-ATGTTAAAAGAACGGGTAACTGC-3’	

Check_Acm1_UP	5’-TTCGCTGTCGGTGAAAGTGG-3’	TR012
Check_Acm1_DO	5’-TATTTTCGTTATCGCCGTATACC-3’	
Check_Lys1_UP	5’-TAAGTCAGCGACAATGTATTCGG-3’	TR013
Check_Lys1_DO	5’-ATGGCGGGAATAAACACTCG-3’	
Check_LytH_UP	5’-ATTCCTTAACCAATCCGTTG-3’	TR014
Check_LytH_DO	5’-TATCAAAACCACCAAAAATCG-3’	
Check_LytA_UP	5’-TTGAAAATCGGTGTGTGAAC-3’	TR006
Check_LytA_DO	5’-TACATTCGTTAAAATAACAGACC-3’	
Check_LytB_UP	5’-ACAAGTACTCAACAATATGC-3’	TR015
Check_LytB_DO	5’-TATTTAAGCACTCGGTATTC-3’	
Check_LytD_UP	5’-AACTTACGACAAAAGGGTGTGG-3’	TR016
Check_LytD_DO	5’-AACACGGTCTTGAATGACGCCC-3’	
Check_MltA_UP	5’-ATGCCCCGATAGATATATTCC-3’	TR017
Check_MltA_DO	5’-TTCTGATAGTGCTGCTAAGGC-3’	
**Primers used for the sequencing of deletion vectors**		
85	5’-GTTTTTTTCTAGTCCAAGCTCACA-3’	pNZ5319
85_compl	5’-TTATTCGTTTGATTTCGCTTTCG-3’	
87_bis	5’-TTGATGATTGGTTCGGAAGGCACG-3’	
87_bis_compl	5’-TATATAGTTTACCCCGTCAGC-3’	
**Primers used for the construction of over-expression vectors**		
Acm2_NcoI	5’-CTTCCATGGGGACAAAAAAAGTAGTAACTT -3’	pGITR010
Acm2_XbaI	5’-TTTTCTAGACCCCTAGCCTTCAAGCTTAGCAACATAGTG-3’	
Lys2_NcoI	5’- CTTCCATGGGTAATTTTAAATCACAAAATCC -3’	pGITR011
Lys2_XbaI	5’-CCCTCTAGATTAGCCAATCTTAGC -3’	
**Primers used for the validation of the acquisition of over-expression vectors**		
pNZ8048_UP	5’-TACTGACAATAGAAACATTAAC-3’	pNZ8048 and derivatives
pNZ8048_DO	5’-TATCAATCAAAGCAACAC-3’	

[a] Restriction sites introduced in the primers are underlined

Table S2. Transcriptomic data of PGH-encoding genes of *L. plantarum* WCFS1 grown in chemically defined medium (CDM) at 28°C (fermentors F12 and F27, mean value) vs. CDM + 300mM NaCl at 28°C (fermentors F6 and F28, mean value), CDM at 37°C (fermentor F18), and CDM + 300mM Nacl + two-fold higher concentration of amino-acids at 37°C (defined here as SCDM conditions) (fermentor F21) (FermDB platform, http://www.cmbi.ru.nl/fermDB, [1])

Gene name[a]	Microarray fold change[a]		
	300mM NaCl	37C°	300mM NaCl, 2 x [aa], 37°CSCDM[b]
acm2 (lp_2645)	1.0	1.1	1.0
***lys2* (lp_3093)**	**1.6**	**2.4**	**3.2**
***acm1* (lp_1138)**	**1.9**	**3.0**	**2.8**
lys1 (lp_1158)	1.1	1.1	1.1
***lytH* (lp_1982)**	**0.2**	**0.4**	**0.2**
lytA (lp_3421)	1.0	1.1	1.1
lytB (lp_2162)	1.0	1.0	1.0
***lytC* (lp_2520)**	**1.6**	**1.5**	**1.9**
***lytD* (lp_1242)**	0.9	1.0	**2.1**
mltA (lp_0302)	1.3	1.2	1.1
***mltB* (lp_3014)**	**1.7**	**1.6**	**1.8**
***mltC* (lp_3015)**	**0.2**	**0.6**	**0.4**

[a] Genes for which the expression is ≤ or ≥1.5 fold repressed or induced are indicated in bold.
[b] SCDM, stress chemically defined medium, stress conditions used in this work for phenotypic characterization of PGH-deficient strains.

Table S3. Differences in muropeptide composition resulting from *N*-acetylglucosaminidase and γ-D-Glu-mDAP muropeptidase activities between *L. plantarum* NZ7100 (WT) and TR0010 ($Acm2^{-}$) or TR006 ($LytA^{-}$).

Peak[a]	Proposed structure[b]	Observed m/z	Calculated[c] $[M+Na]^+$	Acm2[-d]	LytA[-d]
**3**	**Tetra missing GlcNAc**	759.31	759.35	**ND**	
5	*Di*	720.25	720.29		*Down*
8	*Tri-D-ALa-mDAP(NH_2)*	1133.56	1133.53		*Down*
15	*Di (Ac)*	762.20	762.30		*Down*
18	*Tri-D-ALa-mDAP(NH_2) (Ac)*	1175.52	1175.54		*Down*
20	*Tetra-D-ALa-mDAP(NH_2) (Ac)*	1246.50	1246.57		*ND*
21	*Tri-D-ALa-mDAP(NH_2) (Ac)*	1175.48	1175.54		*ND*
**23c**	**Tri-Tetra missing GlcNAc**	1609.86	1609.75	**Down**	
25	*Tri-Tetra-D-ALa-mDAP(NH_2)*	2054.77	2054.95		*ND*
27b	*Tri-D-ALa-mDAP (NH_2) (2Ac)*	1217.51	1217.55		*ND*

[a] Peak numbers and structures were previously assigned for *L. plantarum* NZ7100 [2].

[b]Tri, disaccharide tripeptide (L-Ala-D-iGln-mDAP(NH_2)); Tetra, disaccharide tetrapeptide (L-Ala-D-iGln-mDAP(NH2)-D-Ala); Disaccharide, GlcNAc-MurNAc; (NH_2), amidation; Ac, acetylation on MurNAc or GlcNAc.

[c]Sodiated molecular ions were the most abundant ones on Maldi-Tof mass spectra for all muropeptides.

[d] The muropeptides that have changed in abundance in Acm2- and LytA-deficient strains compared to strain NZ7100 (WT) are bold and italics, respectively. Their identity or their absence was checked by Maldi-Tof mass spectrometry analysis of the peaks of the mutant chromatograms. Symbols: ND, not detected in the mutant compared to WT; Down, reduced amount of at least 50%.

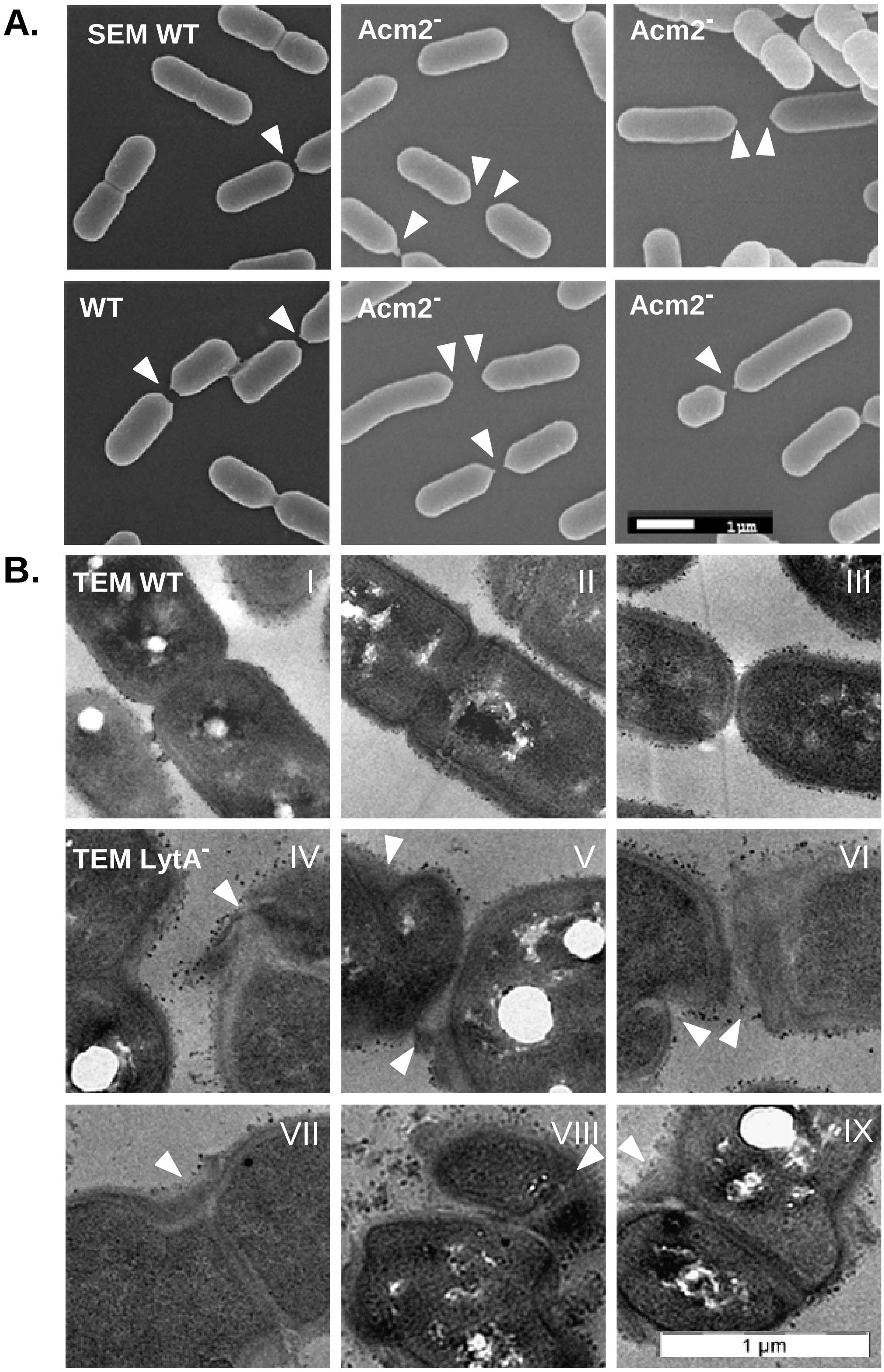

FIG. S1. Cell separation defects of Acm2$^-$ and LytA$^-$ mutant strains compared to NZ7100 (WT, control). A. Micrographs obtained by scanning electron microscopy (SEM) showing more pointed poles (white arrows) after cell separation for Acm2$^-$ mutant cells compared to WT cells. B. Transmission electron microscopy (TEM) showing altered septum formation (white arrows) for LytA$^-$ mutant cells (IV, V, VI, VII, VIII, and IX) compared to WT cells (I, II, and III). Growth conditions as defined in the legend of Fig. 2.

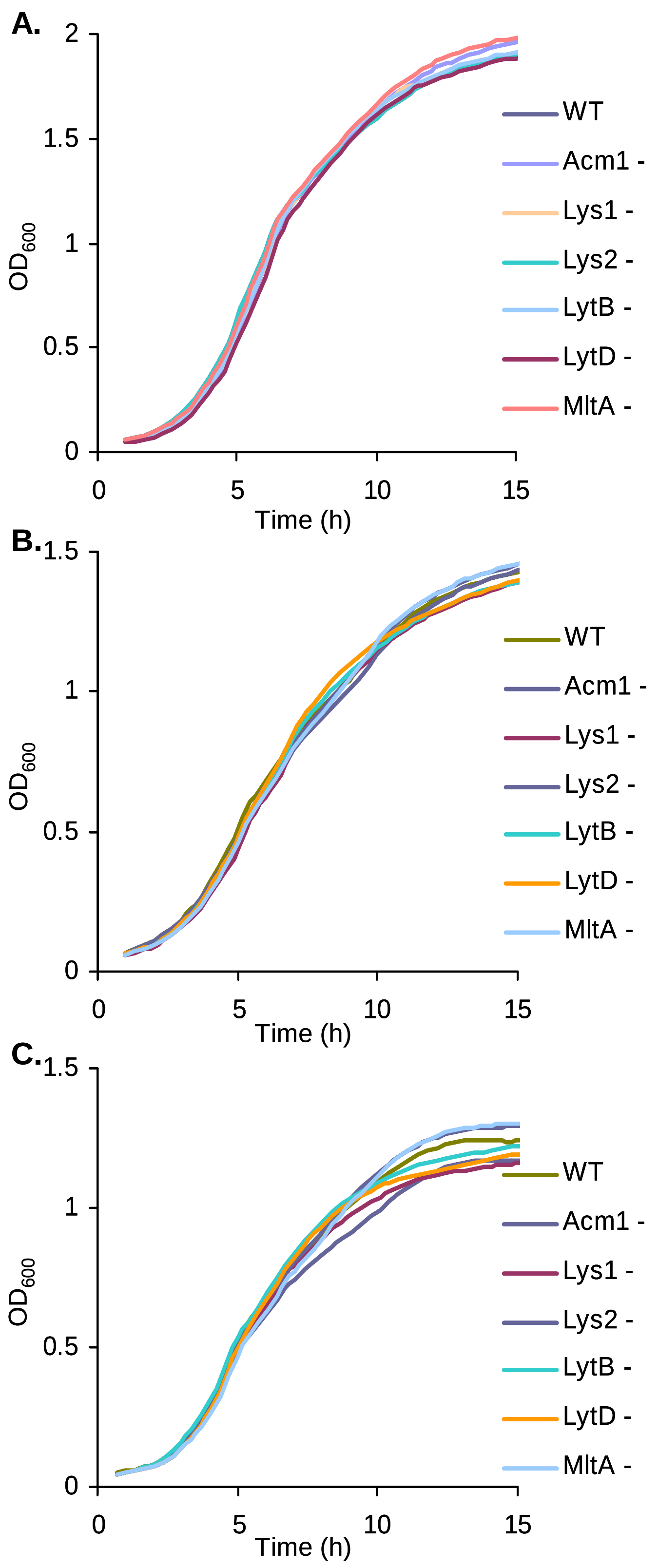

FIG. S2. Growth curves of *L. plantarum* NZ7100 (WT), *acm1* mutant (Acm1[-]), *lys1* mutant (Lys1[-]), *lys2* mutant (Lys2[-]), *lytB* mutant (LytB[-]), *lytD* mutant (LytD[-]), *mtlA* mutant (MtlA[-]), in MRS (A), CDM (B) and SCDM (C). Mean values (n=3).

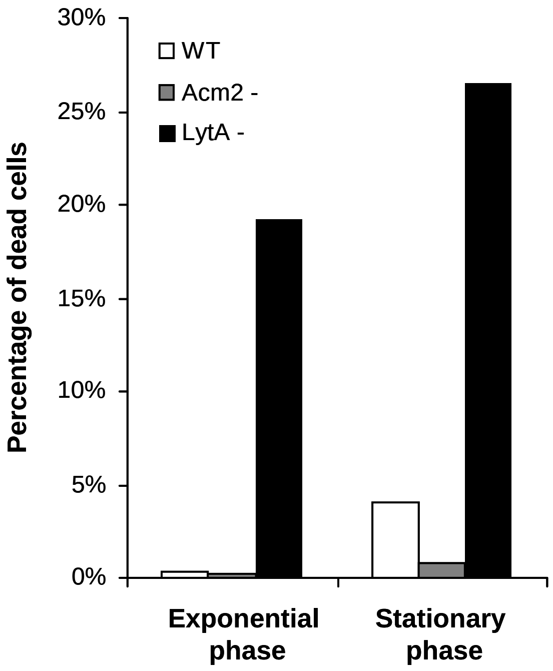

FIG. S3. Effect of *acm2* and *lytA* mutations on cell integrity. Percentage of dead cells was monitored in MRS medium in the exponential (6 h) and stationary (16 h) growth phases for *L. plantarum* NZ7100 (WT), Acm2^{-}, and LytA^{-}. Cell counting was performed by epifluorescence microscopy using propidium iodide (red; labels damaged cells) and SYTO-9 (green; labels all cells). The percentage of damaged cells labeled with propidium iodide was calculated with respect to the total number of cells labeled with SYTO-9. Enumeration was done for a minimum of 300 cells for each strain.

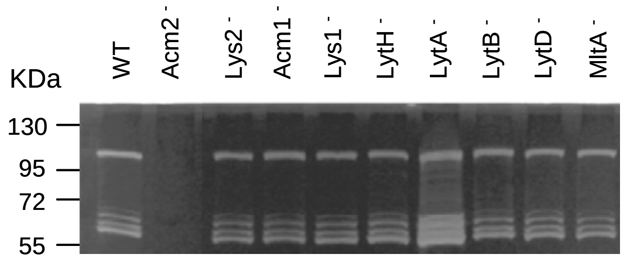

FIG. S4. Zymogram with cell extracts of *L. plantarum* NZ7100 (WT), *acm2* mutant (Acm2^{-}), *lys2* mutant (Lys2^{-}), *acm1* mutant (Acm1^{-}), *lys1* mutant (Lys1^{-}), *lytH* mutant (LytH^{-}), *lytA* mutant (LytA^{-}), *lytB* mutant (LytB^{-}), *lytD* mutant (LytD^{-}) and *mltA* mutant (MltA^{-}) against dead cells of WT.

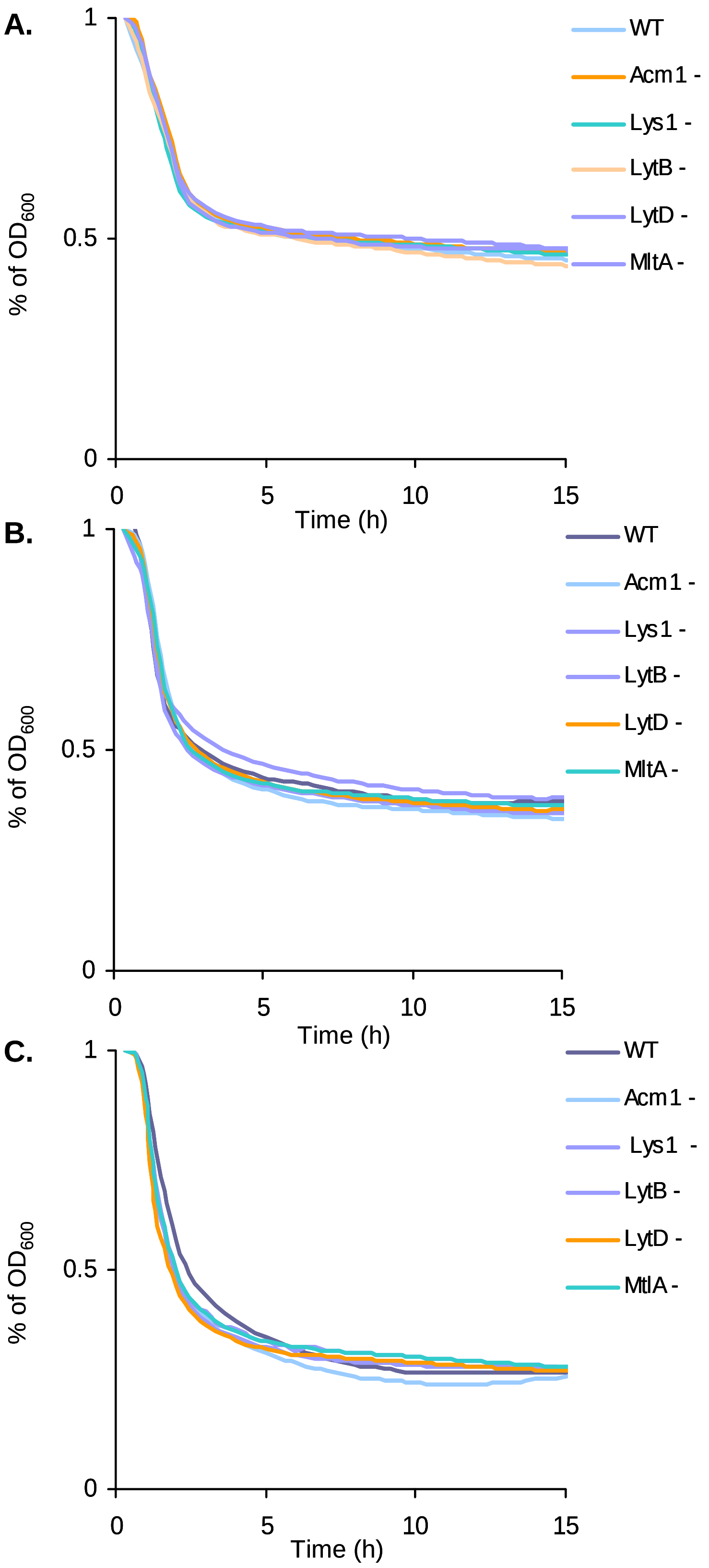

FIG. S5. Autolysis curves in presence of Triton X-100 (0.05%)of *L. plantarum* NZ7100 (WT), *acm1* mutant (Acm1$^-$), *lys1* mutant (Lys1$^-$), *lytB* mutant (LytB$^-$), *lytD* mutant (LytD$^-$), *mtlA* mutant (MtlA$^-$), in MRS (A), CDM (B) and SCDM (C). Mean values (n=3).

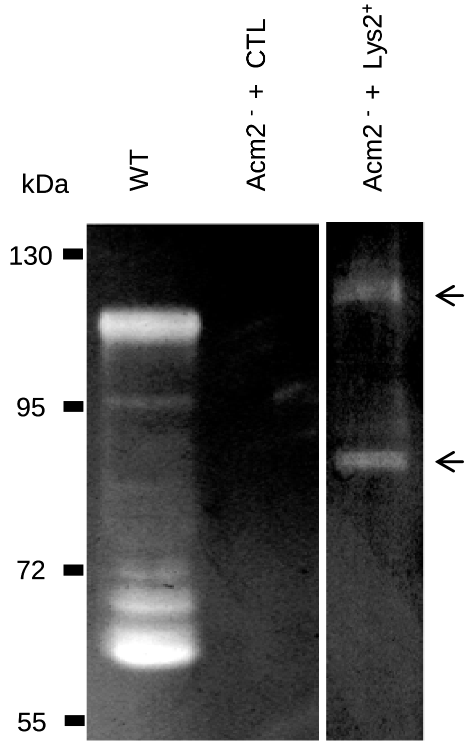

FIG. S6. Zymogram with cell extracts of *L. plantarum* NZ7100 (WT), *acm2* mutant carrying the empty plasmid pNZ8048 (Acm2$^-$ + CTL), and *acm2* mutant overexpressing *lys2* (Acm2$^-$ + Lys2$^+$) grown in presence of the nisin inducer in MRS against dead cells of WT.

REFERENCES

1. Bron PA, Wels M, Bongers RS, van Bokhorst-van de Veen H, Wiersma A, Overmars L *et al.*: **Transcriptomes reveal genetic signatures underlying physiological variations imposed by different fermentation conditions in *Lactobacillus plantarum*.** *PLoS One* 2012, **7:** e38720.

2. Bernard E, Rolain T, Courtin P, Guillot A, Langella P, Hols P *et al.*: **Characterization of O-acetylation of N-acetylglucosamine: a novel structural variation of bacterial peptidoglycan.** *J Biol Chem* 2011, **286:** 23950-23958.
